# Cancer Clocks Out for Lunch: Disruption of Circadian Rhythm and Metabolic Oscillation in Cancer

**DOI:** 10.3389/fcell.2016.00062

**Published:** 2016-06-24

**Authors:** Brian J. Altman

**Affiliations:** ^1^Abramson Family Cancer Research InstitutePhiladelphia, PA, USA; ^2^Abramson Cancer CenterPhiladelphia, PA, USA; ^3^Division of Hematology-Oncology, Department of Medicine, University of Pennsylvania Perelman School of MedicinePhiladelphia, PA, USA

**Keywords:** circadian rhythm, oncogenes, metabolism, cancer metabolism, molecular clock, oscillation, gene expression regulation

## Abstract

Circadian rhythms are 24-h oscillations present in most eukaryotes and many prokaryotes that synchronize activity to the day-night cycle. They are an essential feature of organismal and cell physiology that coordinate many of the metabolic, biosynthetic, and signal transduction pathways studied in biology. The molecular mechanism of circadian rhythm is controlled both by signal transduction and gene transcription as well as by metabolic feedback. The role of circadian rhythm in cancer cell development and survival is still not well understood, but as will be discussed in this Review, accumulated research suggests that circadian rhythm may be altered or disrupted in many human cancers downstream of common oncogenic alterations. Thus, a complete understanding of the genetic and metabolic alterations in cancer must take potential circadian rhythm perturbations into account, as this disruption itself will influence how gene expression and metabolism are altered in the cancer cell compared to its non-transformed neighbor. It will be important to better understand these circadian changes in both normal and cancer cell physiology to potentially design treatment modalities to exploit this insight.

## Introduction: The circadian clock controls gene expression and metabolism

The majority of eukaryotes possess a circadian clock to optimize gene expression and metabolism to the day-night cycle. Cancer cells may disrupt normal circadian oscillation to release cells from control of gene expression and metabolism and provide a growth advantage. In mammals, many familiar processes such a sleep/wakefulness, feeding, blood pressure, and body temperature are synchronized by the circadian clock (Millar-Craig et al., 1978; Spiteri et al., 1982; Cagnacci et al., 1992; Bass, 2012). The “central clock” is governed by blue-light sensing in the eye and subsequent processing in the hypothalamic suprachiasmatic nucleus (Moore and Eichler, 1972; Liu et al., 1997; Ruby et al., 2002), while “peripheral clocks,” which will be the focus of this Review, are present in virtually all organs and individual cells in the body, and are synchronized by the central clock through signals such as hypothalamic-pituitary-directed release of adrenal corticosteroids, but can also operate independently of central clock input (Buijs et al., 1999). Peripheral clocks are strongly entrained by the time of feeding, and misalignment of feeding and the central clock has recently been shown to lead to metabolic syndrome (Mukherji et al., 2015a,b). Synchronization of the peripheral clock can be simulated in cell culture by treatment with the corticosteroid dexamethasone (Balsalobre et al., 2000), or the simple act of changing culture media (Yeom et al., 2010), and thus, circadian oscillations are likely common in most non-transformed cells lines and many cancer lines as well.

**Figure 1:**
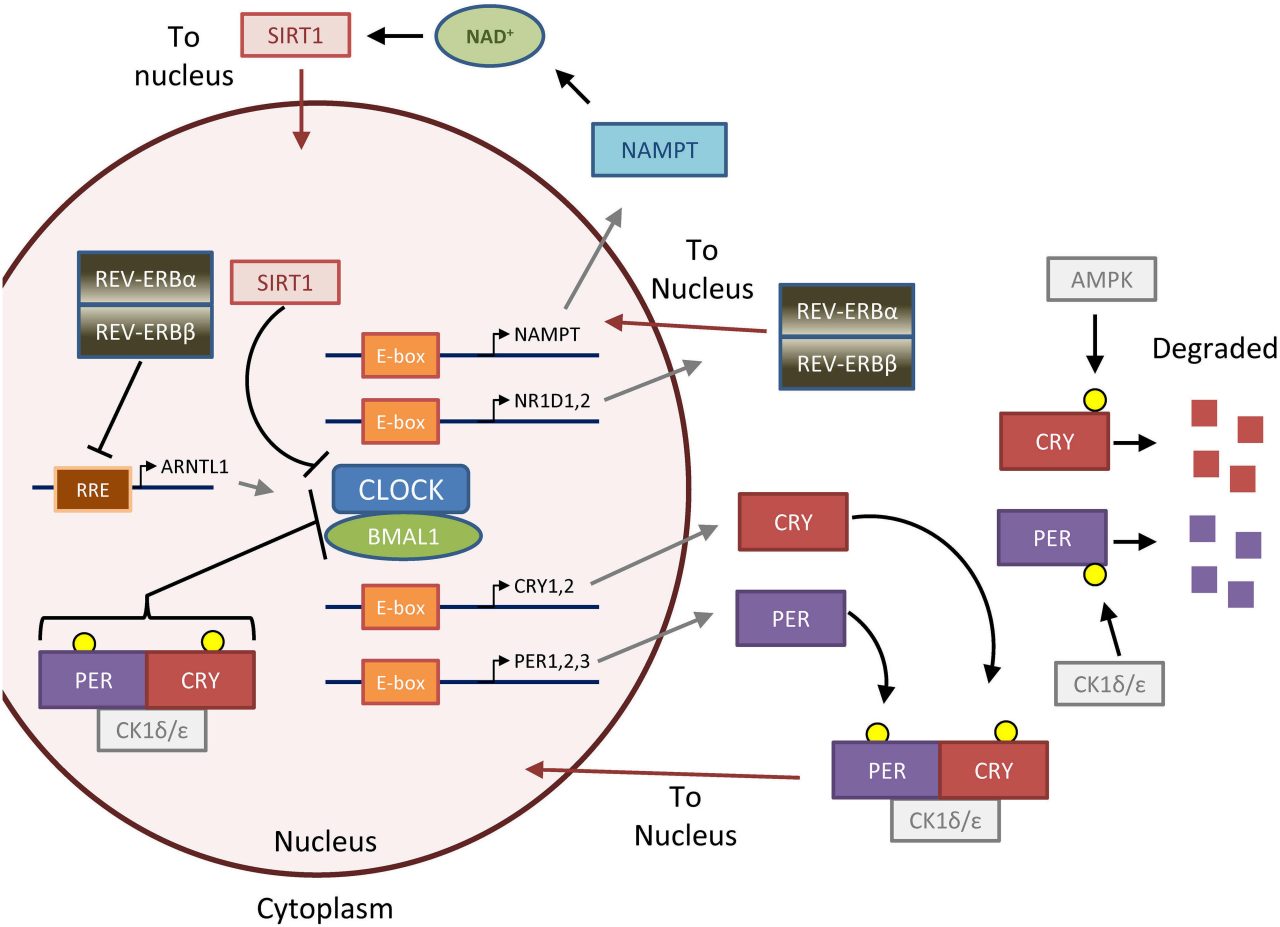
**The feedback loops that form the molecular clock**. The molecular clock is controlled by the master transcription factor heterodimer CLOCK-BMAL1, which is regulated by two major negative feedback loops that generate 24-h oscillation of clock activity and target genes (Gallego and Virshup, 2007; Mohawk et al., 2012). In the first and most important loop, CLOCK-BMAL1 upregulates *PER* and *CRY* through binding to E-box DNA elements. Unbound PER and CRY proteins are phosphorylated by casein kinase 1 ε/δ (CK1ε/δ) and AMPK (AMP-kinase), respectively, to lead to degradation. GSK3 (glycogen synthase kinase 3, not pictured) can also phosphorylate PER and CRY to promote their degradation (Harada et al., 2005; Iitaka et al., 2005). Otherwise, PER and CRY form a complex with CK1, which translocates to the nucleus to repress CLOCK-BMAL1 activity. PER and CRY are then eventually degraded in a CK1-dependent manner (not pictured), and the time delay in the first loop forms an approximately 24-h cycle which is particularly dependent on dynamics of PER regulation (D'alessandro et al., 2015). In the second loop, CLOCK-BMAL1 upregulates the negative transcription factors REV-ERBα and β (gene names *NR1D1 and NR1D2*) and the positive transcription factors RORα,β, or γ (not pictured), which repress or activate BMAL1 (gene name *ARNTL*) transcription, respectively, through binding to RRE (R-response element) DNA sequences. The importance of this second loop is underscored by the fact that mice lacking REV-ERBα and β, which form a complex and act together, lack normal circadian gene oscillation in the liver (Bugge et al., 2012; Cho et al., 2012). Several accessory loops exist; in one that will be highlighted in this review, SIRT1 (sirtuin 1) deacetylase tunes CLOCK-BMAL1 activity by opposing the histone acetyl-transferase (HAT) activity of CLOCK (Asher et al., 2008; Nakahata et al., 2008, 2009; Ramsey et al., 2009). SIRT1 is regulated by the metabolite NAD, which in turn is produced by the NAD-salvage enzyme NAMPT (nicotinamide phosphoribosyltransferase), the rate-limiting enzyme of the NAD salvage pathway involved in NAD recycling and synthesis from dietary nicotinamide or niacin. Together, these primary and accessory loops lead to the 24-h expression of target genes and oscillation of downstream metabolic processes. Figure reprinted and modified from Altman et al. (2015), with permission from Elsevier.

The molecular circadian clock is governed by several feedback loops (Figure 1) that lead to 24-h oscillations of target gene expression, defined by their *amplitude* (height)*, phase* (position), and *period* (length). Several well-described and detailed mathematical models of this molecular oscillation exist, which have been used to make predictions about perturbations of the molecular clock (Leloup and Goldbeter, 2003; Relogio et al., 2011; Hirota et al., 2012; Kim and Forger, 2012). The best-characterized organ with respect to circadian rhythm is liver, where more than 20% of mRNAs oscillate (Panda et al., 2002; Storch et al., 2002; Ueda et al., 2002; Koike et al., 2012). In the whole mammal, up to 50% of protein-coding RNAs and 30% ofnon-coding RNAs oscillate in at least one organ, with the liver, kidney, and lung being the most “circadian”; however, there is little overlap in circadian gene expression between organs, with only 10 genes oscillating in all examined cell types (Zhang et al., 2014). Ribosome occupancy of mRNA and protein translation also demonstrate rhythmicity (Jang et al., 2015; Janich et al., 2015; Lipton et al., 2015), and thus, circadian rhythm strongly controls gene expression and translation, though the specific identity of oscillating genes may vary.

Circadian control of metabolism has been extensively studied on the level of organs. Many specific metabolites, including lipids, amino acids, and glycolytic intermediates, oscillate in mouse liver and human blood, saliva, and even breath (Dallmann et al., 2012; Eckel-Mahan et al., 2012; Kasukawa et al., 2012; Martinez-Lozano Sinues et al., 2014). Anabolic pathways in liver, including nucleotide biosynthesis and ribosomal biogenesis, also showed circadian oscillation (Fustin et al., 2012; Jouffe et al., 2013). On the other hand, appreciation of the oscillation of metabolism on a cell-autonomous level (as observed in tissue culture) is just becoming appreciated. Two studies demonstrated that NAD (nicotinamide adenine dinucleotide) oscillates in cell culture and liver (Figure 1) (Nakahata et al., 2009; Ramsey et al., 2009), which controls rhythmic mitochondrial oxidation (Peek et al., 2013). More recently, we observed in U2OS osteosarcoma cells, a commonly used model of circadian rhythm, that intracellular glucose showed circadian oscillation (Altman et al., 2015). This finding is supported by another study showing oscillation of NADH/NAD+ ratio in epidermal stem cell culture, which may reflect oscillation in glucose metabolism (Stringari et al., 2015). An unbiased metabolomic analysis is still needed to determine the extent of cell-autonomous metabolic oscillations.

Metabolism itself may also control the clock. Several nearly-simultaneous studies uncovered that the NAD- and NAMPT-regulated deacetylase SIRT1 opposes the acetylytansferase activity of CLOCK protein activity (Doi et al., 2006) to deacetylate PER2, BMAL1, and histones, leading to alterations in both phase and amplitude of circadian gene oscillation (Asher et al., 2008; Nakahata et al., 2008, 2009; Ramsey et al., 2009). NAD availability may also influence circadian rhythm through regulation of PARP (poly-ADP-ribose polymerase) to regulate CLOCK-BMAL1 protein and DNA binding (Asher et al., 2010). Emerging evidence suggests that glucose availability may affect circadian rhythm, in part by contributing to O-GlcNAcylation of PER2 to control its activity (Kaasik et al., 2013; Oosterman and Belsham, 2016). It has long been observed that cancers have altered metabolism (Warburg, 1956; Vander Heiden et al., 2011; Stine and Dang, 2013), and that many cancers may have disrupted circadian rhythm (Levi et al., 2008); however, the significance and mechanism of the circadian dysrhythmia in cancer are poorly understood.

## Oncogenic alteration of circadian rhythm

Mutations in molecular clock genes, including promoter methylation, coding region mutation, deletion, or rare amplification, have been documented at a low frequency (less than 20% incidence per tumor type) across many different types of cancer (Cerami et al., 2012; Savvidis and Koutsilieris, 2012; Gao et al., 2013; Uth and Sleigh, 2014). Given that these mutations disrupt normal oscillation, it has been suggested that the clock may be tumor suppressive. Many proto-oncogenes and tumor suppressors are normally under circadian control (Sahar and Sassone-Corsi, 2009), and so disruption of oscillation could potentially release these proteins to be constitutively overexpressed or suppressed. This Review will focus on several notable examples of oncogenic pathways that are often mutated in cancer and have a well-described relationship to circadian rhythm. Given the frequency of mutation in the pathways detailed below, it can be speculated that many cancers with these and perhaps other oncogenic mutations have altered or disrupted circadian rhythm and altered oscillation of gene expression and metabolism.

## RAS

The RAS family of GTP-ases (H-, K-, and N-RAS) is mutated in many cancers to constitutively activate their GTPase function and hyperstimulate downstream mitogen-activated kinase (MAPK) signaling. Oncogenic RAS is known to promote transformation and altered cell metabolism (Pylayeva-Gupta et al., 2011; Kimmelman, 2015), and work spanning decades suggests that wild-type RAS is both influenced by and influences the circadian clock, and thus, mutated oncogenic RAS may potentially alter circadian rhythm. RAS is highly conserved among lower organisms in *Animalia*, and it was shown in *Drosophila* that RAS and the MAPK signaling family mediated circadian rhythm, and inversely that the MAPK pathway itself was governed by circadian oscillation (Williams et al., 2001). Further studies in *Drosophila* revealed that ERK (a critical downstream target of RAS) could directly phosphorylate CLOCK and thus increase the output of clock-controlled genes (CCGs) (Weber et al., 2006). Similarly, clock-controlled genes were increased by active RAS in the bread mold *Neurospora crassa* (Belden et al., 2007). In mammals, RAS and downstream MAPK signaling oscillate in neurons and in the liver, suggesting circadian control in both the central and peripheral clocks (Tsuchiya et al., 2013; Serchov et al., 2016). Neuronal constitutively activated RAS dramatically disrupted circadian gene oscillation and mouse circadian activity through upregulation of CCGs, in a pathway that was dependent on downstream activity of GSK3β (Serchov et al., 2016), and another study further implicated RAS in disruption of CCGs downstream of GSK3 (Spengler et al., 2009). As discussed in the Figure 1 legend, GSK3 is a regulator of CRY and PER stability. While little work has been done to demonstrate this mechanism in cancer, one recent study identified mutated RAS as a mediator of circadian rhythm disruption in colon cancer cells, potentially through upregulation of *CRY1* (Relogio et al., 2014). Thus, while strong evidence exists in multiple organisms and model systems that active RAS can alter circadian rhythm, specifically by upregulating CCGs, the potential role in cancer cell metabolism and physiology remains unclear.

## LKB1/AMPK

The AMP-kinase (AMPK) is an ancient protein complex conserved in nearly all eukaryotes that responds to metabolic stress (Hardie, 2014) by sensing increases in the AMP:ATP ratio, and inhibiting biosynthetic processes while upregulating catabolic metabolism to restore ATP levels (Hardie and Alessi, 2013). The chief upstream kinase responsible for phosphorylating and activating AMPK downstream of metabolic stress, LKB1 (liver kinase B1), is mutated or lost in many cancers, including up to 35% of non-small-cell lung carcinomas (Shackelford and Shaw, 2009). Thus, AMPK may function as a tumor suppressor in some cancers, and indeed, AMPK-promoting compounds such as the widely used complex-I inhibitor metformin and related biguanides have been investigated in preclinical and clinical models (Pollak, 2012).

AMPK plays a strong role in controlling circadian rhythm, and regulates the clock by directly phosphorylating and promoting the degradation of CRY1 (Lamia et al., 2009), and promoting the degradation of PER2 through CK1ε activation (Eide et al., 2005; Um et al., 2007), which both lead to upregulation of CCGs; however, whether this led to a shortening or lengthening of the period was unclear. Underscoring the importance of CK1ε downstream of AMPK, metformin was shown to upregulate *Csnk1* (protein CK1) isoforms in the mouse and alter oscillation of circadian and metabolic genes (Barnea et al., 2012). In a separate pathway, AMPK increases NAD+ levels to activate SIRT1, leading to additional clock modulation (Fulco et al., 2008; Canto et al., 2009; Um et al., 2011; Brandauer et al., 2013). Cancer treatments that activate AMPK, including metformin or anti-metabolic therapies such as the lactate dehydrogenase A inhibitor FX11 (Le et al., 2010), would be expected to alter the molecular clock in affected cells. Strikingly, loss of either LKB1 or of both catalytic subunits of AMPK completely abrogated circadian oscillation, even in the absence of metabolic stress, in several models such as MEFs or mouse liver (Lamia et al., 2009; Um et al., 2011). This raises two interesting possibilities: first that AMPK is an integral accessory regulator of the circadian clock, and second, that cancers deficient in AMPK activity through loss of LKB1 may have a deficient clock.

## p53

The p53 tumor suppressor protein is mutated or lost in a large number of cancers, leading to dysregulation of metabolism, cell cycle, and apoptosis (Berkers et al., 2013; Chen, 2016). Recent evidence suggests an interdependent relationship exists between p53 and PER2, which has fascinating implications for circadian rhythm and metabolism. PER2 may directly regulate p53 activity: inactivation of PER2 by mutation delayed p53 accumulation after ionizing radiation, sensitizing mice to both cancer development and death (Fu et al., 2002). Supporting these data, two studies showed that high levels of PER2 in cancer cell lines and glioma xenografts correlated with increased p53 induction and apoptosis (Hua et al., 2006; Zhanfeng et al., 2016). However, the possible molecular mechanism of p53 activity regulation by PER2 was not well described in these studies.

This relationship is bidirectional, as p53 can influence PER2 both at the gene expression and protein level. p53 can antagonize *PER2* expression by directly binding to the *PER2* promoter and blocking CLOCK-BMAL1 transactivation of the gene (Miki et al., 2013). Either loss of p53 or accumulation of p53 protein caused phase shifts in mouse circadian behavior, suggesting that both basal and induced p53 can regulate the clock through *PER2* modulation. Adding another layer of complexity, two complementary studies demonstrated that PER2 protein can form a dimer with p53 in the cytoplasm to stabilize p53 and allow translocation to the nucleus, either under basal conditions or genotoxic stress (Gotoh et al., 2014, 2015). Once in the nucleus, PER2-p53 also binds its E3 ubiquitin ligase MDM2 (mouse double minute 2 homolog), and this trimeric complex prevents p53 ubiquitination and degradation, allowing for increased transactivation of p53 targets. The authors hypothesized that PER2 may exist in two pools: one bound to p53, and one bound to CRY and CK1ε for control of circadian rhythm and subsequent degradation (Gallego and Virshup, 2007).

Several interesting conclusions can be made from the above findings. First, given that PER2 strongly controls p53 gene expression, stability, and localization, and that PER2 levels oscillate in the cell, wild-type p53 protein and activity itself must oscillate, making these cells more or less sensitive to DNA damage at certain times. p53 mRNA and protein oscillation was observed in several studies (Horiguchi et al., 2013; Miki et al., 2013), and in fact, circadian sensitivity to p53 activity was demonstrated in several older studies that identified circadian variation in radiation toxicity in rodents (Pizzarello et al., 1964; Lappenbusch, 1972). However, it remains unclear whether oscillation of p53 activity was due to *TP53* mRNA oscillation, or oscillation of the upstream E3 ubiquitin ligase MDM2 to control p53 protein stability (Horiguchi et al., 2013). Since p53 feeds back to suppress *PER2* expression and alter protein localization, the above pathway may be an as-of-yet uncharacterized accessory loop of endogenous clock control. Additionally, it has been appreciated in recent years that DNA damage induces oscillatory p53 activity and protein levels, with a period of about 6 h and dependent on phosphorylation of both p53 and MDM2 (Lahav et al., 2004; Geva-Zatorsky et al., 2010). It is likely that, after DNA damage, this inherent p53 oscillation, circadian control of p53, and p53 control of PER2 interact in some significant way, but this has not yet been studied.

Another upshot of this relationship is that altered p53 status should disrupt circadian oscillation. DNA damage and other insults induce and stabilize p53 (Chen, 2016), and p53 can control circadian rhythm through its modulation of *PER2* transcription, protein stability, and protein localization (Miki et al., 2013; Gotoh et al., 2014, 2015), so it can be hypothesized that under stress p53 induction will dramatically alter the circadian clock through its modulation of PER2, which may perhaps be an adaptive pro-survival process. On the other hand, p53 mutation loss or mutation in cancer would dramatically affect circadian rhythm, both by allowing for increased *PER2* gene expression (Miki et al., 2013) and by altering the availability of PER2 protein to bind to other partners such as CRY (Gotoh et al., 2014, 2015). One interesting question is how mutant p53 that has acquired novel DNA-binding and transactivation functions would affect *PER2* and circadian rhythm (Muller and Vousden, 2013). Thus, loss or mutation of p53 in cancer may alter or disrupt circadian rhythm, with unknown consequences to cancer physiology.

## MYC

The *MYC* and related *MYCN* oncogenes (encoding MYC and N-MYC) are translocated, amplified, or mutated in many cancers, and can dramatically upregulate genes involved in glucose and glutamine metabolism, ribosomal, lipid, and nucleotide biogenesis, and cell cycle progression (Stine et al., 2015). Given that MYC recognizes and binds to E-Box DNA promoter elements identical to those recognized by CLOCK-BMAL1, it was theorized that CLOCK-BMAL1 could bind to MYC target genes (Fu et al., 2002; Fu and Lee, 2003), an idea later borne out by observation that CLOCK-BMAL1 could inhibit N-MYC-dependent gene transactivation (Kondratov et al., 2006). Given that the *MYC* gene itself contains multiple E-box elements (Battey et al., 1983), it was shown that CLOCK-BMAL1 regulates endogenous *MYC* circadian oscillation and oscillation of MYC-target genes, both by direct BMAL1 binding to the *MYC* promoter, as well as by additional translational and posttranslational control by the molecular clock machinery (Fu et al., 2002, 2005; Okazaki et al., 2010; Repouskou et al., 2010; Repouskou and Prombona, 2016). It is also likely that endogenous MYC influences the clock, but this potential role has not been elucidated.

Given that MYC rewires the cell for altered metabolism and growth, we hypothesized that hyperactivated oncogenic MYC could disrupt the molecular clock and thus alter circadian oscillation of metabolism. We found that overexpressed MYC and N-MYC upregulated many clock family members, including PER2, CRY1, and most notably, REV-ERBα (Altman et al., 2015), leading to a dramatic suppression of BMAL1 expression and oscillation, which could be rescued by knockdown of REV-ERBα and its binding partner REV-ERBβ (Bugge et al., 2012; Altman et al., 2015). Our study also showed that oncogenic MYC dramatically altered and disrupted circadian oscillation of glucose and AMPK phosphorylation (Altman et al., 2015), thus suggesting that oncogenic mutation may disrupt circadian gene expression, metabolic oscillations, and oscillation of cellular bioenergetics.

Interestingly, MYC alteration of circadian gene expression seems to be highly cell-type specific. For instance, a recent study in HEK-293 and colon cancer cells showed that overexpressed MYC bound the *PER1* promoter exclusively, and rather than transactivating expression, this binding led to a *downregulation* of *PER1* due to competitive inhibition of CLOCK-BMAL1 promoter occupancy, which would presumably also lead to circadian disruption (Repouskou and Prombona, 2016). Alternately, MYC overexpression in embryonic stem cells led to PER cytoplasmic accumulation rather than upregulation (Umemura et al., 2014). Another study identified *CSNK1e* (protein CK1ε) as a synthetic lethal target of MYC and N-MYC upregulated in neuroblastoma and other human cancers (Toyoshima et al., 2012), and upregulation of CK1ε would be expected to destabilize the clock through its promotion of PER degradation and activation of BMAL1 (Gallego and Virshup, 2007). It remains to be determined in what contexts overexpressed MYC in cancer deregulates clock genes through either promoter co-occupancy, competition with CLOCK-BMAL1 to trasactivate or repress target genes, or through forming novel complexes with either CLOCK or BMAL1. Nonetheless, all of the above studies documented a role for overexpressed MYC in disruption of circadian oscillation, which as we showed has consequences for metabolic oscillation and cell physiology (Altman et al., 2015).

## Conclusions and perspectives: Connections between oncogenic mutation, metabolism, and circadian rhythm, with an eye toward chronotherapy

**Figure 2:**
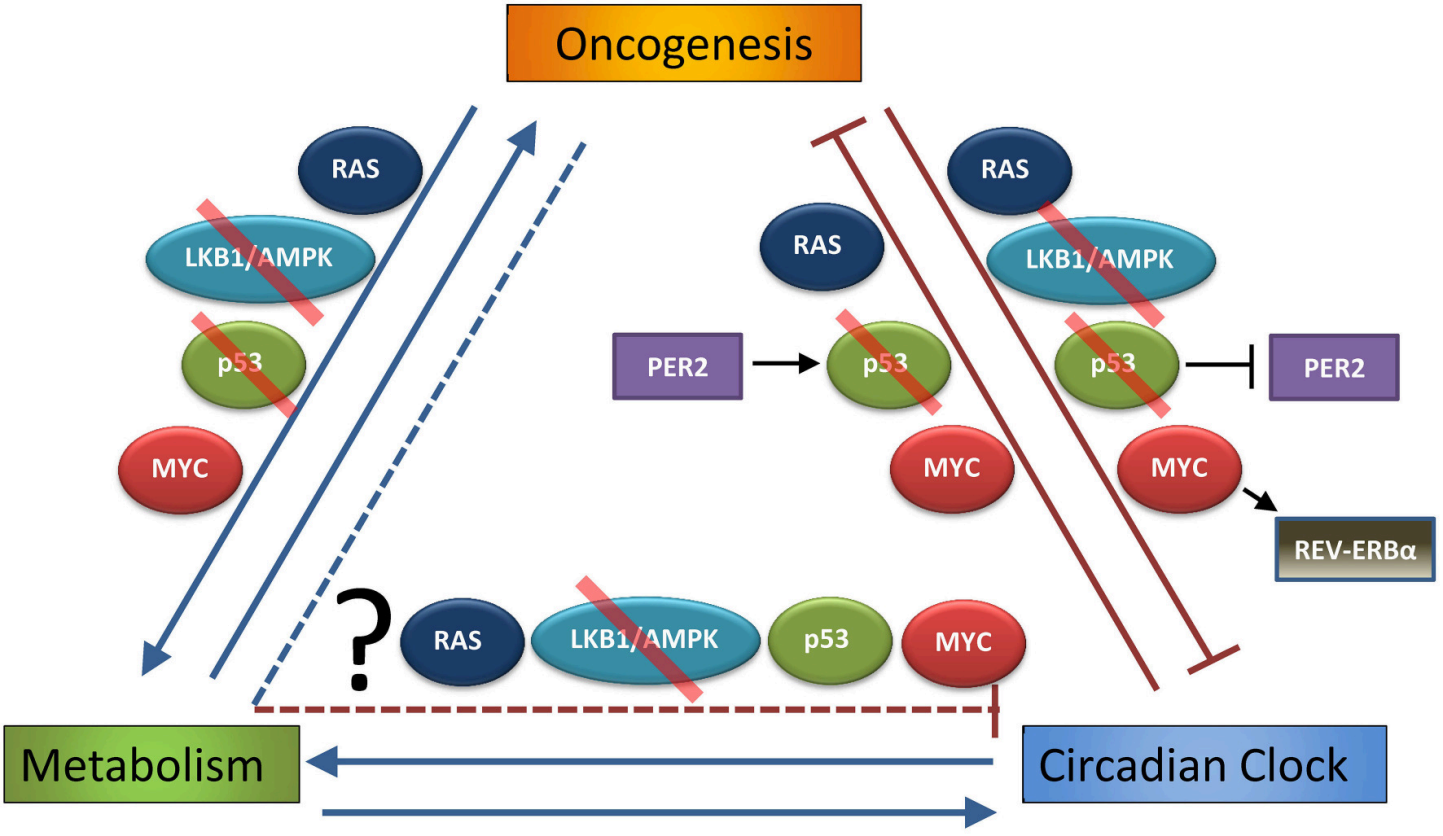
**Interdependent relationship of oncogenesis, metabolism, and the circadian clock**. Oncogenesis (defined as hyperactivation of pro-growth pathways downstream of mutations or alterations in RAS or MYC, or loss of normal function in growth-suppressive pathways as p53 or LKB1/AMPK, that lead to uncontrolled cell growth and transformation) is well known to alter cell metabolism, and these metabolic changes are necessary to support oncogenesis (Hirschey et al., 2015). As discussed in the Introduction, circadian rhythm strongly influences metabolism, and several metabolic pathways can feed back to control circadian rhythm. This Review demonstrates that oncogenic pathways, such as RAS, LKB1/AMPK, p53 (in part through p53 regulation of PER2), or MYC (in part through MYC activation of REV-ERBα), may disrupt or alter the normal peripheral circadian clocks of organs and individual cells. On the other hand, it has been shown that endogenous RAS, p53 (through PER2 regulation), and MYC oscillate on the genetic and functional level, and so it has been suggested that the clock itself is tumor suppressive (by regulating these oncogenes and tumor suppressors) and thus can prevent oncogenesis. What is still unknown is the extent to which altered metabolism downstream of cancer (and pathways such as RAS, LKB1/AMPK, p53, and MYC) contributes to suppression of the molecular clock. Red slash indicates pathways and proteins that are often lost in cancer, making them tumor-suppressive pathways.

Circadian rhythm is an essential part of cell physiology that underlies many biological processes. Common pathways involved in oncogenesis alter the molecular clock through a diverse set of mechanisms, and RAS, p53, and MYC are strongly regulated by the circadian machinery, suggesting a deep interdependent relationship that is lost when these genes are altered in cancer. The manner by which circadian oscillation is altered is varied: active RAS causes increases in amplitude, p53 loss causes phase shifts, and MYC seems to cause a suppression of overall oscillation. Adding another layer of complexity, both oncogenic alterations and circadian rhythm regulate metabolism, and metabolism itself can feed back to control circadian rhythm. An interesting consequence is that oncogenic alterations can potentially disrupt circadian rhythm both through direct effects on gene expression and protein regulation, and also through alteration of metabolism (Figure 2). However, the potential role of altered cancer metabolism in disruption of circadian rhythm has not been addressed. Additionally, it is not clear how potential oncogenic alterations of circadian rhythm respond to or modify synchronizing signals from the central clock.

Several unanswered questions arise from the work reviewed here. First, *why* do many cancers potentially disrupt circadian rhythm? One can imagine that circadian oscillation, which imposes a “rest” phase every 24 h, is maladaptive to cancer cells, and so altering or destroying this rhythm might allow transformed cells to outcompete their non-transformed neighbors. The clock may be upstream of normal tumor suppressors and proto-oncogenes (Sahar and Sassone-Corsi, 2009) to regulate normal metabolism and growth, and as shown above, these pathways seem to form feedback mechanisms with the clock that are lost in cancer, perhaps releasing oncogenes, tumor suppressors, and even metabolism from circadian control.

Second, *how* can the cancer research community use this knowledge of circadian disruption to better treat cancer? The answer may lie in chronotherapy, or timed administration of treatment to patients, based on circadian rhythm, to increase efficacy and reduce toxicity of drugs or radiation. Dozens of traditional cancer therapeutics, including the anti-metabolite folate pathway antagonist methotrexate, have known circadian-dependent toxicity (Levi et al., 2010). Excitingly, recent research indicates that several targeted therapies currently in clinical use have strongly circadian-dependent efficacy depending on the time of day given, including but not limited to erlotibin (inhibits EGFR, used in lung cancer), lapatinib (inhibits HER/Neu and EGFR, used in breast cancer), and evirolimus (inhibits mTOR, used in some breast cancers and pancreatic neuroendocrine tumors), and in fact there are several chronotherapy dosing schedules under clinical trial (Dallmann et al., 2016). Better knowledge of how specific oncogenes disrupt normal oscillation of tumor cells could lead to more effective strategies in delivery of targeted or metabolic therapies. Circadian disruption is potentially an essential part of the evolution of cancer, and further study will allow us to better understand both the benefits to cancer of this disruption, and how this knowledge can be used to help patients.

## Author contributions

BA conceived of the review topic, performed the literature research for the review, wrote the review and designed the figures, and edited the review for final submission and revision.

### Conflict of interest statement

The author declares that the research was conducted in the absence of any commercial or financial relationships that could be construed as a potential conflict of interest. The reviewer RS and handling Editor declared their shared affiliation, and the handling Editor states that the process nevertheless met the standards of a fair and objective review.
